# Bayesian Network analysis of piglet scours

**DOI:** 10.1038/s41598-017-06399-2

**Published:** 2017-07-24

**Authors:** Benjamin J. J. McCormick, Lechelle K. Van Breda, Michael P. Ward

**Affiliations:** 1 Unaffiliated, Insch, Aberdeenshire AB52 6UE Scotland; 2 Sydney School of Veterinary Science, The University of Sydney, Camden, Australia

## Abstract

Diarrhoeal disease (scours) in piglets, often associated with enterotoxigenic *Escherichia coli* (ETEC), is a substantial financial burden to the pig industry worldwide. Previous research has not explicitly examined the relationships between farm, pen and microbiological factors. Here we present a state of the art analysis to reveal empirical indirect – as well as direct – associations between management factors as putative risks for scours in pre- and post-weaned piglets. A Bayesian Network is constructed to identify the optimal structural model describing the relationships between risk factors. An additive model is then built to estimate more epidemiologically familiar odds ratios. Farm-level variance dominates the model, making many pen-level associations null. However, there is evidence that pre-weaning scours are less likely on farms with <400 sows (0.14, 0.03–0.50). Our results strongly suggest that smaller production units (piglets/pen) could reduce the incidence of scours in piglets. There is also some evidence that ownership of other livestock is a potential risk factor for pre-weaning scours, although this was observed only at one farm. Future research should be directed at better understanding the role of herd size and investigating the relationship between managing other livestock and the occurrence of scours in pig herds.

## Introduction

Pre- and post-weaning diarrhoea (scours) is a major production-limiting disease in Australian pig farms costing an estimated $AU7 million each year^1^. Outbreaks of scours are common and result in reduced growth rates, higher medication costs, increased morbidity and mortality^2^. Despite the importance of scours, the management practices that may contribute to or affect disease outbreaks are not well understood in Australian pig production systems.

As a syndromic disease, the causes of scours are many and varied. Stressors such as alterations to the quality, type or quantity of the diet, poor air quality and other environmental factors can contribute to scours^3–6^. Enterotoxigenic *Escherichia coli* (ETEC) have the ability to colonise the lower intestine via fimbrial adhesins (F4 (K88), F5 (K99), F6 (987P), F18 and F41)^7^ and the production of enterotoxins (heat-stable STa and STb, heat-labile LT, and Stx2e)^8^ results in dehydration and scouring. At weaning, maternal antibodies wane and with limited acquired immunity, piglets are susceptible to pathogenic bacterial species and more vulnerable to developing scours. Once piglets are placed in a pen following weaning, bacteria are shed in the faeces and this can then act as a source of infection to other piglets in the same pen or via shared housing. The survival and spread of bacteria is largely due to management practices once the pathogen is present on the farm.

More than three decades ago a pivotal study of weaning disorders in 89 French pig herds with contemporary conventional husbandry characterised disease onset as an eco-managerial problem^9^. Through examination of 515 environmental variables, 10 primary risk factors for weaning disorders were identified^5^. This study crucially recognised that these risks were interlinked and context dependent. Later the predictive power of individual (putative risk) factors were shown to vary over time^10^.

Australian herd management strategies have evolved since the early studies of environmental drivers of scours. For example, housing has changed with weaner piglets generally housed in larger groups and raised in eco-shelters on deep litter^11, 12^. Similarly, sow stalls are being phased out in favour of grouped housing, there are improvements to diet and nutrition and management of diseases through enhanced biosecurity procedures, and increased vaccine availability^13^. These changes have increased productivity, but have not been matched by comparable research into the drivers of diarrhoeal disease and whether key risk factors have changed^14^.

Here we use state of the art analytical techniques to explicitly examine the causation of scours in the context of pig farm management. Using a survey of pig farms in southeastern Australia, we construct a Bayesian Network (BN) using structure discovery algorithms to reveal empirical relationships between production factors and pre- and post-weaning scours^15^. Traditional analytic approaches reduce disease systems to a single outcome and ignore the relationships between risk factors that can lead to indirect risk pathways. When translating such results into applied tools, ignoring indirect pathways can overestimate the success of interventions; furthermore, understanding the whole disease system can lead to novel interventions through the control of highly influential factors that act as junctions of risk pathways^15^. To address multi-causal disease syndrome, traditional approaches to understand disease causation are inadequate.

## Results

Characteristics of the farms included in the analysis are shown in Table 1. Herd size ranged from 45 to 20,000 sows per farm (mean 721 sows). Most farms were observed to have either (or both) pre- and post-weaning scours (17% and 24%, respectively) at the time of sampling. Approximately 22% of pens had a history of diarrhoea, as reported in the questionnaire. The number of pens with <199 and ≥200 piglets (the median value) was similar, although the distribution was skewed by one herd with very large pens (1,000 piglets).

**Table 1:** Data from a questionnaire survey of 17 farms and sampling of 174 pens of piglets used as inputs to an additive Bayesian network following filtering of variables for missing data or significant associations with diarrhoea, pre- or post-scours.

Variable	Number of pens	
	No	Yes
History of diarrhoea (y/n)	135	39
Suspected beta-haemolytic (>0%)^a^	61	113
Suspected non beta-haemolytic (<100%)^a^	159	15
Average weaning age (weeks) (≤5)	42	132
No litter (y/n)	150	24
Number of Sows (<400)	76	98
Indoor intensive production (y/n)	73	101
Number of buildings (≤5)	108	66
Other livestock (y/n)	27	147
Number of piglets/pen (<200)	92	82
Number of piglets/shed (<300)	88	86
Pre-weaning scours (y/n)	12	162
Post-weaning scours (y/n)	42	132
*E*. *coli v*accination of Sows (y/n)	47	127
Pre-weaning *E*. *coli* vaccination (y/n)	153	21
Weaner feed additives/acids (y/n)	66	108
Antibiotics in water (y/n)	114	60
Recent disease (y/n)	83	91
Small or medium pen sizes (y/n)	53	121
Number of buildings (≤5)	114	60
Ventilation (y/n)	23	151
Controlled temperature weaner pens (y/n)	35	139
Anaerobic effluent disposal (y/n)	46	128
**Proportion**	**Beta**-**haemolytic** ***E***. ***coli***	**Non**-**beta**-**haemolytic** ***E***. ***coli***
0	74 (38.0%)	**Nil**
0.1	**2** (**1**.**0%**)	**Nil**
0.2	**22** (**11**.**3%**)	**Nil**
0.3	**4** (**2**.**1%**)	**Nil**
0.4	**14** (**7**.**2%**)	**Nil**
0.5	**5** (**2**.**6%**)	**Nil**
0.6	**12** (**6**.**2%**)	**6** (**3**.**1%**)
0.7	**3** (**1**.**5%**)	**1** (**0**.**5%**)
0.8	**15** (**7**.**7%**)	**9** (**4**.**6%**)
0.9	**2** (**1**.**0%**)	**1** (**0**.**5%**)
1	**42** (**21**.**5%**)	178 (91.3%)

The two variables included in the model were: 1. beta haemolytic *E*. *coli* (>0%); and 2. non-beta haemolytic *E*. *coli* (<100%). These variables reflect the ‘unusual’ occurrence, as indicated by the bolding above. In the case of non-beta haemolytic *E*. *coli*, detection was near ubiquitous (91% positive). For beta-haemolytic *E*. *coli* the distribution was more complex, but polarised towards all (21.5%) or none (38%). Therefore rather than categorising beta-haemolytic *E*. *coli* based on 50% presence it was partitioned as some/none with none used as the reference category i.e. the odds of at least some beta-haemolytic *E. coli* present in the pen.

^a^ *E*. *coli* detections were modelled based on the number of pens with a given proportion of 1. beta-haemolytic *E*. *coli* and 2. non-beta haemolytic *E*. *coli*, as follows (data shown is prior to application of filtering procedures which reduced the number of pens from 195 to 174).

**Figure 1:**
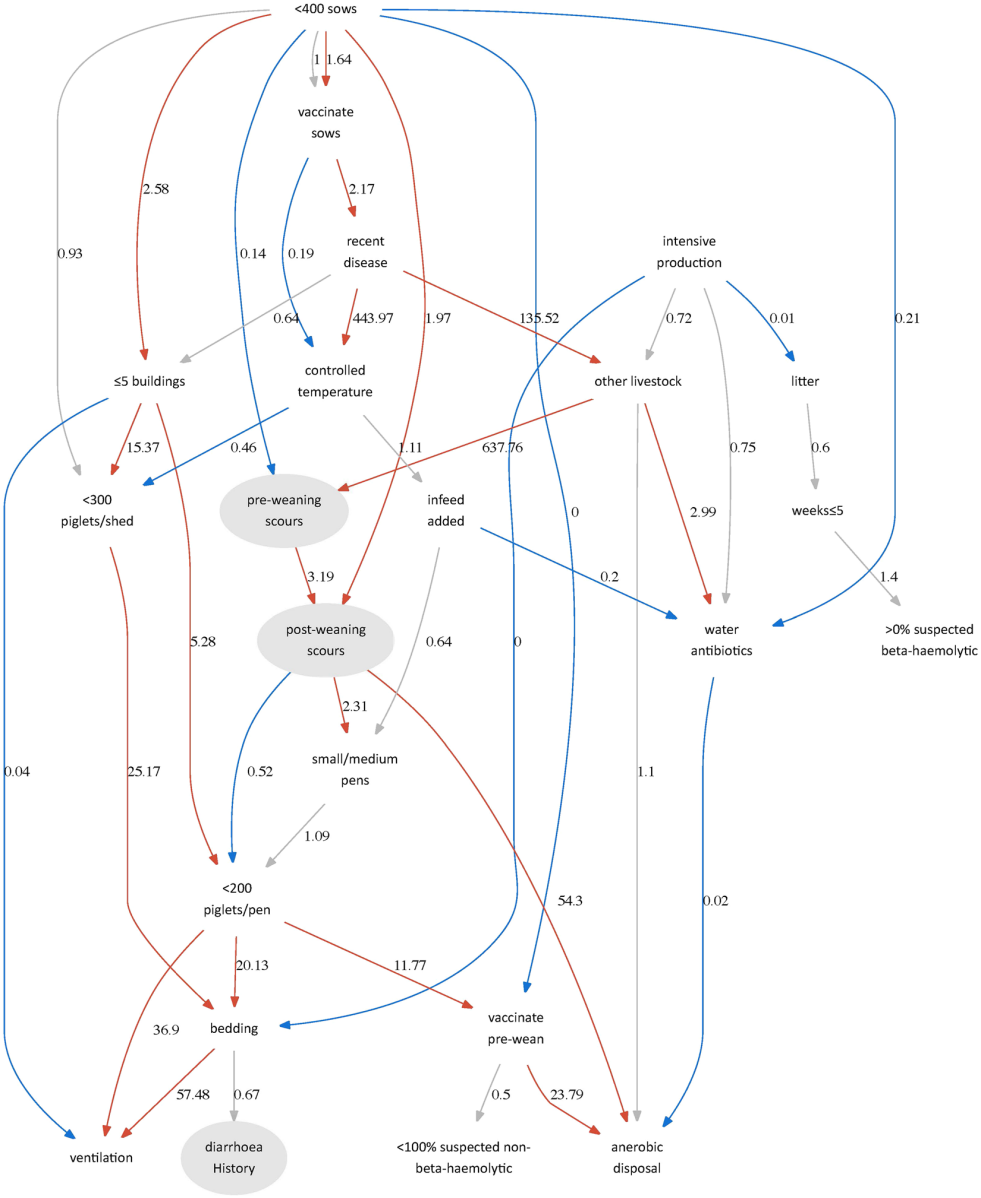
Additive Bayesian Network showing odds ratios of observing each a given value of a node conditional on the network. The three diarrhoeal variables are highlighted in grey. In this figure, the network includes fixed effects only having identified the structure via non-parametric bootstrapping and a tabu modified greedy hill-climbing search for the optimal structure. Red arcs indicate positive associations that do not include 0 in the 95% credibility interval; blue arcs are negative associations that no not include 0 in the 95% credibility interval; the 95% credibility interval of grey arcs include 0.

The identification of the BN structure assumed conditional probability tables for each node. In converting the model to an additive BN, it was necessary to remove intercept terms because in most cases these were collinear with other variables. Removal of the intercept terms made the model tractable. The model structure – averaged over the bootstrap samples – included 43 arcs with greater than 50% support (Fig. 1). Estimating odds ratios from the additive BN reduced the number of arcs with statistical support to 30. Up to 10% of data was missing due to producers declining to answer questions.

The risk of pre-weaning scours was a function of the number of sows kept, whereby ownership of <400 sows was protective against pre-weaning scours (median odds 0.14, and 95% credibility interval 0.03–0.50). The odds ratio of pre-weaning scours was perfectly predicted by ownership of other livestock because only one farm had neither other livestock nor pre-weaning scours. This arc was therefore removed after model fitting (a dashed line in the figure). Out of the 17 farms included in the final analysis, 14 (82%) had both ownership of other livestock and evidence of pre-weaning scours.

Pre-weaning scours were a risk factor for post-weaning scours (3.19, 1.99–5.34). There was strong evidence that the number of sows was associated with post-weaning scours; however, unlike pre-weaning scours, a smaller number of sows was a risk factor (1.97, 1.00–3.87). The odds of a history of diarrhoea, whilst related to the presence of bedding material, did not have any statistically supported associations once the additive BN was calculated.

Of note, there was no statistical support for associations between recent disease events (within the last twelve months) and either pre- or post-weaning scours. Also, an association between suspected beta haemolytic *E*. *coli* and either pre- or post-weaning scours was not supported by the data. Beta haemolytic *E*. *coli* identification was a statistical orphan node in the network (*i*.*e*. there was no statistical evidence to support associations between this and any other variable in the BN). Non-beta haemolytic *E*. *coli* was also an orphan variable, although it appeared (with weak evidence) to occupy a very different set of pathways.

**Figure 2:**
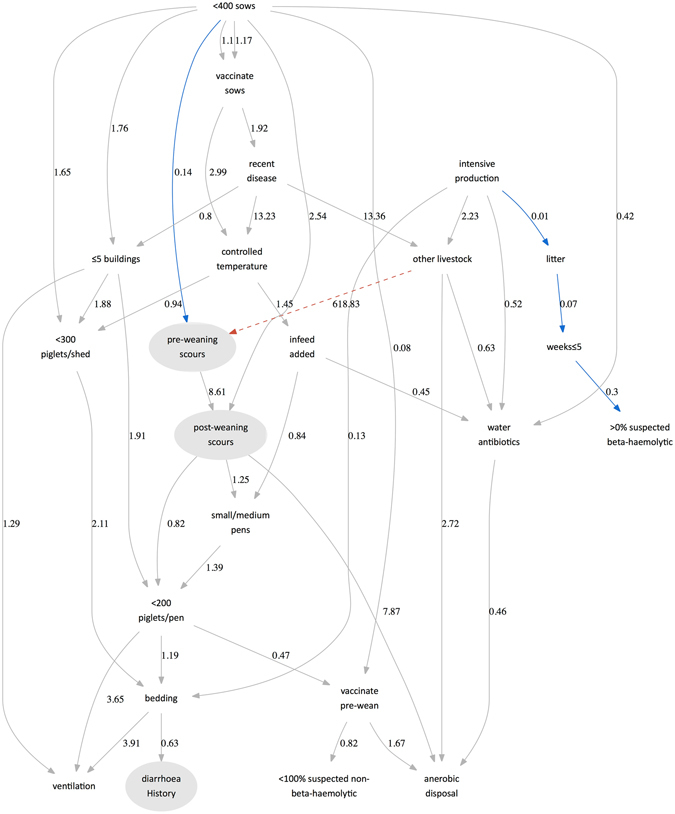
Additive Bayesian Network showing odds ratios of observing each a given value of a node conditional on the network. The three diarrhoeal variables are highlighted in grey. In this figure, the network includes both fixed effects and random effects for each farm. The model structure was identified via non-parametric bootstrapping and a tabu modified greedy hill-climbing search for the optimal structure. Red arcs indicate positive associations that do not include 0 in the 95% credibility interval; blue arcs are negative associations that no not include 0 in the 95% credibility interval; the 95% credibility interval of grey arcs include 0. The arc between ownership of other livestock and pre-weaning scours was manually removed because of separation in the data (at the farm level) (dashed line).

The inclusion of farm-level random effects dramatically reduced the number of supported arcs (from 30 to 5, shown in Fig. 2). This is important because it suggests that many factors, perhaps unsurprisingly, co-occur based on management practices. Importantly, there was continued evidence for the associations between the number of sows and pre-weaning scours. This suggests that even after accounting for clustering within farms, there is strong evidence to support this association at the pen level. The association between livestock and pre-weaning scours was removed because it reflected a single farm and was therefore likely to be biased.

Curiously, the other arcs that were supported with the inclusion of farm-level random effects (i.e. the odds of suspecting >0% beta haemolytic *E*. *coli*) had not previously been supported by the credibility intervals of the additive BN. This is surprising because at the pen level, ignoring the clustering within farms, this factor was not statistically supported. However, the inclusion of farm clustering revealed stronger evidence that the odds of detecting beta haemolytic *E*. *coli* were reduced by weaning at >5 weeks of age (0.30, 0.08–0.97), which itself was reduced by the absence of litter. The odds of not having litter were strongly associated with not having intensive indoor production, suggesting that beta-haemolytic *E*. *coli* may be more likely to be seen in extensive production (although this association was not significant; χ^2^ = 0.88, *p* = 0.35, and hence no direct arc was supported in the BN).

## Discussion

Scours in weaning pigs is an on-going challenge to pig production in Australia. Despite considerable investment in counter measures, little research has been done to untangle management factors that may reveal the eco-epidemiology of diarrhoeal diseases. Here we explicitly examine the relationships between farm and pen level factors that are putative risk factors for scours.

The risks of pre-weaning scours were robustly predicted by the number of sows. This implies a route of transmission of enteropathogens that is worthy of further investigation. On larger farms – indicated by a higher number of sows (and the arc from post-weaning scours to the number of piglets per pen and pen size) – pre-weaning scours was more likely to be observed and these farms, at least some, were at risk of post-weaning scours. The latter association is, however, more likely to be a farm management issue than a piglet-pen risk. In particular, it is interesting that the presence of beta-haemolytic *E*. *coli* and the use of vaccination – in either sows or pre-weaned piglets – had no direct associations with pre- or post-weaning scours. Beta-haemolytic *E*. *coli* can be detected in the faeces of both healthy and diarrhoea piglets but studies have observed higher levels in diarrhoeal piglets^6, 16^; vaccination is a common practice in pig production^14^.

Recently a Canadian study assessing biosecurity practices and diseases in pigs found strong associations between proximity to other livestock and disease transmission^17^. Although Cox *et al*. targeted Porcine reproductive and respiratory syndrome, Swine influenza, *Mycoplasma* pneumonia and Swine dysentery, and used a proximity measure (whereas we asked specifically “Do you run other livestock on the same property?”), this suggests that by minimising such risk factors associated with *E*. *coli* scours producers could also minimise other diseases known to have dramatic impacts on pig production. We found that only one farm had neither ownership of other livestock nor pre-weaning scours, making it difficult to draw robust conclusions. However, ownership of other livestock could be an indicator of inadequate biosecurity, or might increase the risk of pathogenic strains of *E*. *coli* being introduced to a pig herd. This risk factor is worthy of further investigation. It is a common practice for Australian pig producers to raise other livestock on their farm (van Breda *et al*.^14^).

Though statistically biased (due to separation in the data), the majority of farms analysed had both other livestock on the same farm and pre-weaning scours. This is suggestive of transmission between animals^18^. Once within a pen, smaller management units tend to be less prone to post-weaning scours, however this depends on the management context of a production system^19^. It would be useful to examine alternative causes of scours given that vaccination in general, and for *E*. *coli* in particular, was only tangentially associated with diarrhoeal disease in piglets. Given the plethora of infectious causes of enteropathy it is possible that less ubiquitous species are transmitted between hosts.

A key finding presented here is the dominance of farm-level variability. Transmission routes likely vary between farms, and given the associations at a pen level, are associated with pen and unit size. Fewer piglets per pen are likely to be associated with reduced risk of post-weaning scours. We hypothesise that this is related to the chance of identifying and responding to early signs of scours in a timely fashion, in addition to the differences in management between smaller and larger enterprises. For example, farms with fewer buildings (≤5) are less likely to have ventilation, and pens with ventilation are more likely to have fewer than 200 piglets per pen and to have bedding.

This study is the first to collect so much data on farm, pen and microbiologic aspects of scours from south eastern Australian pig production. As such is it uniquely positioned to explore the balance of management factors that may influence the risk of scours in weaning pigs. However, the dominance of farm-level variability in management factors limits extrapolation of findings to other farms that have different farm practices. The diversity of farm management systems necessitated some computational simplifications (such as binary categorisation of factors). The distribution of farm sizes reflects the breadth of production systems with 80% of pens containing fewer than 350 piglets, but the remaining 20% ranging up to 1000 piglets per pen.

The transient nature of scours makes identifying key risk factors difficult. Bayesian network analysis has been used previously to identify risk factors associated with pig diseases and climate^20^ and antimicrobial resistance in *E*. *coli* ^21^. Bayesian network techniques are an invaluable modern tool to understand and map risk factors and disease. Future work on scours to examine on-farm biosecurity procedures and potential routes and causes of infection would be useful. The effectiveness of control strategies – and particularly the use of antibiotics – for scours in pig production could be explored using Bayesian network techniques.

## Materials and Methods

### Data

Data on 58 variables were collected through a survey of 195 pig pens from 22 farms across southeastern Australia (New South Wales [n = 9], South Australia [n = 3] and Victoria [n = 10]). This survey has been described in detail by Van Breda *et al*.^14^. The number of herds included in this study was based on the available funding and the practicalities of sampling a large geographical area. Farms recruited were identified by swine veterinarians using prior knowledge of *E*. *coli* outbreaks; farms were also recruited at the 2013 Bendigo Pig Fair, Victoria and by contacting farmers directly via industry membership. A preliminary telephone questionnaire was conducted to establish the owner/manager’s willingness to participate and the suitability of their herd to be included in the study. Herds with less than 40 sows were excluded due to the limited number of piglets available for sampling at the time of the herd visit.

A cross-sectional survey was conducted between September 2013 and May 2014 and faecal samples to identify *E*. *coli* were collected from each herd. Samples were stratified to capture piglets one week prior to weaning (10 samples) and piglets that had most recently been weaned on the day of the herd visit (40 samples). Depending on the size of the pen, five samples were collected from pens of <20 piglets; and 10 samples were collected from pens of >20 piglets (an average 8.9 pens [range 1 to 14] were sampled per farm). A formal questionnaire, administered to the owner, manager or the leading farm hand, was conducted once at each farm. It included questions covering management, production and biosecurity practices. Details of the questionnaire, sample management and analysis is described elsewhere^14^. All animal sampling procedures and interactions were carried out in strict accordance with the recommendations made by The University of Sydney Animal Ethics Committee. The protocol was approved by The University of Sydney Animal Ethics Committee (Approval number: N00/7-2013/3/6002).

### Statistical Analyses

BN analyses to address model structural uncertainty are computationally intensive. Therefore, to make the network tractable, all variables were screened to eliminate variables that would either reduce the sample size of the model or had no statistical evidence to justify inclusion in the network. First, the proportion of missing observations for each variable was counted. Fourteen variables with more than 5 per cent of observations missing were dropped from further analysis. This reduced the number of variables from 58 to 44. Second, the remaining variables were examined for univariate association with either pre- or post-weaning scours at the time of herd visit or with a history of diarrhoea using logistic regressions and an inclusive threshold of *P* ≤ 0.2. This was conducted with complete observations and, when the retained variables had also been screened for possible overlap, based on expert knowledge (e.g. the number of samples collected and the percentage *E*. *coli* positive). This resulted in 23 variables with some statistical evidence of association with scours that were retained for further analysis. To construct the BN, observations with missing data for any given variable were excluded, reducing the sample size from 195 to 174 pens and from 22 to 17 farms. The motivation for removing variables during analysis is summarised in Table 2.

**Table 2:** Motivation for removing variables during analysis of a dataset describing scours in 195 pens of pigs on 22 farms in southeastern Australia: (1) due to a proportion of *missing information* >5% that would reduce the power of the Bayesian network model; (2) *no statistical relationship* a P ≤ 0.2 with either diarrhoea or pre- or post-weaning using *all available data* (i.e. some variables had missing observations); (3) *no statistical association* as before after removing any observation that *lacked data* for any variable; (4) removed because of *overlapping interpretation* with other variables retained.

Variable	Reason for removal
Number of production sites	missing information
Land area	missing information
Number of piglets weaned per week	missing information
Pre-wean vaccination	missing information
Town water supply	missing information
Bore water supply	missing information
Natural mating	missing information
Artificial insemination	missing information
Type of flooring	missing information
Frequency of cleaning	missing information
Cleaning using pressure hosing	missing information
Disinfectant use	missing information
Number pigs slaughtered per week	missing information
Truck ownership	missing information
Presence of sow faeces in pre-weaned samples	no statistical relationship – all data
Number of samples collected	no statistical relationship – all data
Maximum beta-haemolytic growth (%)	no statistical relationship – all data
Number of positive non-beta haemolytic samples	no statistical relationship – all data
Type of shelter	no statistical relationship – all data
Max number of piglets per pen	no statistical relationship – all data
Infeed additive antibiotics	no statistical relationship – all data
Infeed additive plasma	no statistical relationship – all data
Infeed additive milk	no statistical relationship – all data
Percentage of beta-haemolytic positive samples	no statistical relationship – restricted data
Genetics sourced from PIC	no statistical relationship – restricted data
Crops grown	no statistical relationship – restricted data
Weaner house grouping	no statistical relationship – restricted data
Farrowing pen type	no statistical relationship – restricted data
Antibiotics administrated to post-weaned piglets	no statistical relationship – restricted data
Age at weaning	no statistical relationship – restricted data
Manure spreading	no statistical relationship – restricted data
Number of positive beta-haemolytic samples	overlapping interpretation
Age of piglets sampled	overlapping interpretation
State	overlapping interpretation
Vaccination of sows	overlapping interpretation

A BN was constructed from the retained variables. Each variable was represented as a binary node and the structure identified using a modified (tabu) greedy hill-climbing search. Structural uncertainty was modelled with non-parametric bootstrapping (1000 samples) and only arcs with greater than 50% support were retained. The conditional-probability model was then translated into a system of mixed-effects logistic regressions. An additive BN has a more epidemiologically familiar interpretation (odds ratios)^22^ with clearer interpretation compared to a BN (with probabilistic contingency tables) and allows the inclusion of random effects representing the 17 farms included in analysis to account for clustering. The disadvantage of an additive BN is the computational limits on examining so many interconnected variables. The conditionally probabilistic model, though conceptually different, was computationally tractable to identify the model structure and allow parameter estimation. Random effects were fit with diffuse gamma distributions. The network structure was calculated by the R package bnlearn^23^ and the parameter estimates in JAGS 3.4.0^24^.

### Data availability

Survey data is available at https://doi.org/10.1371/journal.pone.0172528.s002 and https://doi.org/10.1371/journal.pone.0172528.s003.
